# Improved Upland Cotton Germplasm for Multiple Fiber Traits Mediated by Transferring and Pyramiding Novel Alleles From Ethyl Methanesulfonate-Generated Mutant Lines Into Elite Genotypes

**DOI:** 10.3389/fpls.2022.842741

**Published:** 2022-04-13

**Authors:** Jinesh D. Patel, Sameer Khanal, Rahul Chandnani, Jeevan Adhikari, Nino Brown, Peng W. Chee, Don C. Jones, Andrew H. Paterson

**Affiliations:** ^1^Plant Genome Mapping Laboratory, The University of Georgia, Athens, GA, United States; ^2^Department of Crop and Soil Sciences, The University of Georgia, Tifton, GA, United States; ^3^NESPAL Molecular Cotton Breeding Laboratory, The University of Georgia, Tifton, GA, United States; ^4^Cotton Incorporated, Agricultural Research, Cary, NC, United States

**Keywords:** crop improvement, breeding strategy, fiber quality, allele pyramiding, chemical mutagenesis

## Abstract

Ethyl methanesulfonate (EMS) mutagenesis offers important advantages for improving crops, such as cotton, with limited diversity in elite gene pools. EMS-induced point mutations are less frequently associated with deleterious traits than alleles from wild or exotic germplasm. From 157 mutant lines that have significantly improved fiber properties, we focused on nine mutant lines here. A total of eight populations were developed by crossing mutant lines in different combinations into GA230 (GA2004230) background. Multiple lines in each population were significantly improved for the fiber trait that distinguished the donor parent(s), demonstrating that an elite breeding line (GA230) could be improved for fiber qualities using the mutant lines. Genotypes improved for multiple fiber traits of interest suggesting that allele pyramiding is possible. Compared to midparent values, individual progeny in the population conferred fiber quality improvements of as much as 31.7% (in population O) for micronaire (MIC), 16.1% (in population P) for length, 22.4% (in population K) for strength, 4.1% (in population Q) for uniformity, 45.8% (in population N) for elongation, and 13.9% (in population O) for lint percentage (lint%). While further testing for stability of the phenotype and estimation of yield potential is necessary, mutation breeding shows promise as an approach to reduce the problem of the genetic bottleneck of upland cotton. The populations developed here may also contribute to identifying candidate genes and causal mutations for fiber quality improvement.

## Introduction

Cotton (*Gossypium* spp.) is an important source of natural fiber and oilseed. A total of four Gossypium species were domesticated (Wendel and Cronn, 2003; Pickersgill, 2007; Renny-Byfield et al., 2016) as sources of textile fiber, two Old World diploids (*G. arboreum L* and *G. herbaceum L.*) and two New World tetraploids (*G. hirsutum* and *G. barbadense*) (Brubaker et al., 1999; Wendel and Cronn, 2003). Due to longer and stronger fiber and higher yield, tetraploid cotton species (especially *G. hirsutum*) are now preferred and account for 95% of world cotton fiber production (Wendel et al., 1992; Chen et al., 2007). Cotton breeding is focused mainly on improving yield, fiber quality traits, and developing stress-tolerant varieties (Bradow and Davidonis, 2000; May, 2002). Major fiber quality traits measured by high volume instrument (HVI) or advanced fiber information system (AFIS) are fiber length (LEN), uniformity, strength, fineness, maturity ratio, short fiber content (SFI), and fiber color characteristic [color as reflectance (Rd) and yellowness (+ b)] (Kelly et al., 2012, 2015).

Intensive breeding within a narrow sample of germplasm, together with several decades of focus on transgenic lines, has rendered the elite cotton gene pool; one of the narrowest among the major crops (Lubbers and Chee, 2009) and has been suggested to leave very little room for fiber quality improvement (Van Esbroeck and Bowman, 1998; Iqbal et al., 2001; Paterson et al., 2004; Tyagi et al., 2014). Wild species can provide desirable agronomical traits and be used in the breeding program to broaden the genetic diversity but often carry deleterious alleles. Moreover, reproductive barriers, meiotic drive, hardship in adaptability to new habitats, differences in photoperiodic response, and flowering time complicate selection within crosses involving wild relatives to achieve desired characteristics without reducing yield (Zhang and Percy, 2007; Barb et al., 2014; Waghmare et al., 2016). Cloning of alleles affects fiber traits and transferring them into elite cotton cultivars involves prohibitive time, cost, and regulatory concerns regarding genetically modified organisms (GMOs).

Chemical mutagens such as ethyl methanesulfonate (EMS) that generate single nucleotide “point” mutations offer a means to create new alleles free from problems associated with linkage drag of unfavorable alleles during the cross of wild species. For cotton, one can use EMS to create and screen for mutant lines that have significantly improved fiber qualities as compared to parental lines. In addition, one can expect to find other agronomical traits of interest such as fuzzless seeds, biotic and abiotic stress tolerance, and trichomeless or hairy varieties along with improved fiber quality without affecting the fiber yield (Bechere et al., 2009; Patel et al., 2016). While appreciable screening of a large number of candidate lines is necessary, one might discover germplasm with novel alleles that could contribute to desirable agronomical traits. Several successes in using EMS to enhance elite cotton germplasm have been reported (Auld et al., 2000; Brown et al., 2012; Patel et al., 2014).

In this study, we build on prior work to identify desirable EMS-mutated cotton lines (Patel et al., 2014), transferring novel alleles from their source backgrounds (TAM94L25 and Acala 1517-99) to an elite breeding line, GA2004230. We also investigated the consequences of “pyramiding” such novel alleles in eight populations from different combinations of mutant lines crossed to GA2004230. Significant improvement in fiber qualities of progenies developed by crosses between GA2004230 and selected mutant lines provided motivation to transfer novel alleles from mutant lines to additional elite germplasm and offered support for increased use of EMS mutants for improving cotton fiber quality.

## Materials and Methods

### Plant Sources and Population Development

**FIGURE 1:**
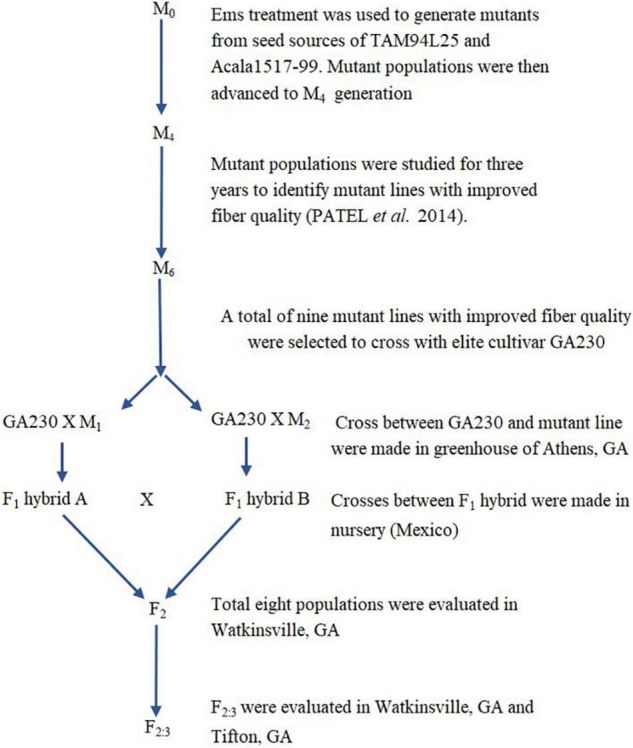
Development of populations for the current research study.

Based on initial screening of large populations of mutant lines generated using EMS in two different genetic backgrounds of *G. hirsutum*, TAM 94L25 (Smith, 2003) and Acala 1517-99 (Cantrell et al., 2000) in Texas (2007) and Georgia (2008), a subset of 157 mutant lines having significant improvements in one or more components of fiber quality, were tested along with control lines at two locations in a replicated trial (Texas and Georgia, 2009). The data from the four environments support the fact that mutant lines with significantly improved LEN, strength, elongation, fineness, lint%, and other quality and yield components can be achieved (Patel et al., 2014). Nine striking mutants were selected from the 157 mutant lines showing improved LEN (one mutant line from Acala 1517-99), fiber elongation (ELON) (one from TAM 94L24), fiber fineness (one each from TAM 94L24 and Acala 1517-99), fiber uniformity (one from Acala 1517-99), fiber strength (STR) (one from Acala 1517-99), lint% (one each from TAM 94L24 and Acala 1517-99), and Rd value (one from TAM 94L25) (Table 1). Each of these nine mutant lines was crossed with a non-transgenic breeding line representing the Eastern Cotton Belt, GA 2004230 (GA230; PVP 201500309) (Lubbers et al., 2006) to develop F1 hybrids in a greenhouse in Athens, GA (Summer, 2012). The F1 hybrids were further crossed to pyramid novel alleles from mutant lines contributing to various fiber traits in GA230 background (Figure 1 and Table 2) in an off-season nursery in Mexico. For comparison, we used M0 parents (TAM 94L25 and Acala 1519-99), GA230, and two additional checks, namely Fibermax 832 (PVP 9800258) and Deltapine 393 (PVP 200400266). The M0 parent serves as an excellent check to demonstrate the effect of transferred novel alleles from the mutant lines on fiber quality as populations developed through these crosses should show significant improvement over the midparent value. Additionally, all parents and checks were replicated ten times in each replication block; thus, planting the mutant lines or the F1 crosses would require more labor, land, and time with little to no change in the outcome.

**TABLE 1:** Mutant lines from Patel et al. (2014) selected for the breeding scheme.

Line #	Mean	Control mean	Parent	Selected for	Percent difference
1903	1.3	1.18	Acala 1517-99	LEN	9.60%
1793	37.1	33.8	Acala 1517-99	STR	9.70%
1524	41.4	38.7	Acala 1517-99	lint%	7.20%
3010	4	4.71	Acala 1517-99	MIC	15.00%
2455	85.9	84.3	Acala 1517-99	UNIF	1.90%
2925	8.68	5.78	TAM 94L25	ELON	50.00%
276	43.1	40.7	TAM 94L25	lint%	5.90%
2877	3.94	4.83	TAM 94L25	MIC	18.40%
1251	80.5	77.4	TAM 94L25	Rd value	4.10%

*MIC: micronaire, LEN: fiber length, UNIF: uniformity index, STR: fiber strength, ELON: fiber elongation, lint%: lint percentage.*

**TABLE 2:** Crossing scheme between F1 hybrid for mutant pyramiding.

Population ID	Crosses between F1 hybrid	Fiber trait targeted	Mutant parental lines	Population size
J	M2925 × GA 230	ELON × MIC	TAM 94L25	100
	M2877 × GA230			
		
K	M1903 × GA230	LEN × STR	Acala 1517-99	98
	M1793 × GA230			
		
L	M3010 × GA230	MIC × lint%	Acala 1517-99	89
	M1524 × GA 230			
		
M	M276 × GA230	Lint% × Rd value	TAM 94L25	45
	M1251 × GA230			
				
N	M2925 × GA230	ELON × Rd value	TAM 94L25	58
	M1251 × GA230			
				
O	M2877 × GA230	MIC × Lint%	TAM 94L25	87
	M276 × GA 230			
				
P	M1930 × GA230	LEN × MIC	Acala 1517-99	97
	M3010 × GA230			
				
Q	M1793 × GA230	STR × UNIF	Acala 1517-99	86
	M2455 × GA 230			

*MIC: micronaire, LEN: fiber length, UNIF: uniformity index, STR: fiber strength, ELON: fiber elongation, lint%: lint percentage.*

### Field Trial and Data Collection

A total of 100 F2 (expected to be 50% donor and 50% GA230 genetic background) progenies from each of these crosses were grown in Watkinsville, GA, (soil type: fine, kaolinitic, thermic typic Kanhapludults) in the year 2013 and were evaluated for fiber quality. Since the populations were equivalent to F2 with respect to the genetic background with 50% donor and 50% GA230 genetic background, the novel mutant alleles affecting the fiber should segregate according to the BC1F1 ratio (since each F1 was heterozygous for each mutant). In 2014, F2-derived F3s with sufficient seeds were planted in a randomized complete block design (RCBD) with two replicates at two locations (Watkinsville and Tifton, GA). The soil type of Watkinsville, GA was Appling coarse sandy loam (fine, kaolinitic, thermic typic kanhapludults) and of Gibbs farm, Tifton, GA was Tifton loamy sand (fine, loamy, siliceous, thermic Plinthic Kandiudult). A total of 35 seeds were planted in plot sizes of 3 m, spaced 1 m apart. Agronomic practices like weeding, irrigation, fertilizer application, and pest management followed local recommendations to commercial growers. Fiber samples obtained from the three environments (Watkinsville, GA, in 2013; Watkinsville, GA, in 2014; and Tifton, GA, in 2014) were ginned using a 20-saw gin (Dennis Manufacturing Inc). Lint weight and seed weight (seed plus fuzz) were measured, and lint% (lint weight × 100/seed cotton weight) was calculated. Fiber properties, namely MIC; LEN, which was measured in inches as upper half mean length; STR measured in grams/tex; ELON in percentage; uniformity index (UNIF) measured in percentage; and short fiber index measured in percentage (SFI), were quantified using the HVI system at Cotton Inc.^1^

### Data Analysis

Data were analyzed using SAS 9.4 (SAS Institute Inc, Cary, NC, United States). The program statement, “Proc CORR” was used to determine the correlations between fiber traits. Narrow-sense heritability of fiber traits was calculated using parent–offspring regression by the SAS “Proc REG” statement. The contribution and significance of genotype, environment, and interaction between genotype and environment for fiber traits were calculated using the SAS statement “Proc GLM.” The total sum of squares (TSS) includes four subsets, attributable to (1) genotype, (2) environment, (3) genotype × environment interaction, and (4) error. The proportion of variable contributions or sum of squares (SS) for genotype, environment, and GXE was the ratio of the respective individual SS to the TSS. Among the total of eight populations, four were developed by crossing mutant lines in TAM 94L25 background to GA230 (populations J, M, N, and O) and four by crossing mutant lines in Acala 1517-99 to GA230 (populations K, L, P, and Q) (Table 2). The mean fiber traits of these populations were compared with the parental means using Fisher’s LSD test at an alpha level of 0.05 to determine the significant improvement in a subpopulation for a fiber trait. The midparent value was calculated as the average of the salient M0 parent (for the population) and GA230. To determine if pyramiding of novel alleles conferring superior fiber traits can produce germplasm with improvement for both fiber traits for which a population was developed (for example, MIC and ELON for population J), Z scores for each line were calculated and the 10 lines with the highest combined Z score for the two fiber traits were compared with parental lines.

## Results

### Heritability Between Generation and Correlation Between Fiber Traits

Narrow-sense heritability was calculated between F2 and F3 generations for each fiber trait based on parent–offspring regression. Moderate heritability for fiber traits was seen in both populations (Table 3). In TAM 94L25 × GA230 (TAGA) populations, LEN (0.43) showed the highest heritability, whereas UNIF (0.17) showed the lowest heritability. Similarly, for Acala 1517-99 × GA230 (ACGA) population, STR (0.44) showed the highest heritability while UNIF (0.27) showed the lowest heritability. Similar heritability values have been observed in other advanced generations and mutant populations (Herring et al., 2004; Chandnani et al., 2017).

**TABLE 3:** Narrow-sense heritability (h2) between F2 and F2:3 value for MIC, LEN, UNIF, STR, ELON, SFC, and LINT% in TAM 94L25 (mutants) × GA230 and Acala 1517-99 (mutants) × GA230 background.

	MIC	LEN	UNIF	STR	ELON	SFI	LINT%

	TAM94L25 (mutants) × GA230						
Watkinsville 2013–2014	0.3	0.45	0.13	0.34	0.41	0.19	0.31
Watkinsville 2013, Tifton 2014	0.48	0.42	0.2	0.26	0.34	0.33	0.35
Watkinsville 2013, average 2014	0.39	0.43	0.17	0.3	0.38	0.26	0.33

	**Acala 1517-99 (mutants) × GA230**						

Watkinsville 2013–2014	0.26	0.36	0.25	0.47	0.32	0.27	0.4
Watkinsville 2013, Tifton 2014	0.39	0.43	0.29	0.4	0.36	0.44	0.43
Watkinsville 2013, average 2014	0.32	0.4	0.27	0.44	0.34	0.36	0.41

*MIC: micronaire, LEN: fiber length, UNIF: uniformity index, STR: fiber strength, ELON: fiber elongation, SFI: short fiber content, lint%: lint percentage.*

Correlation between traits helps to determine if two traits can be simultaneously improved or if improving one trait might impair another trait. Positive correlation generally suggests that two traits can be improved simultaneously, except for correlations involving MIC or SFI, for which negative values are desirable. MIC showed moderate negative correlation with LEN and positive correlation with lint% in both populations and small but significant negative correlation with STR and UNIF in ACGA only. LEN showed a moderate positive correlation with STR and UNIF but a small negative correlation with ELON and lint%. UNIF had a positive correlation with STR and ELONG. STR had a negative correlation with ELONG in TAGA and lint% in ACGA. A moderate positive correlation between ELON and lint% was seen in both populations. SFI had a negative correlation with each fiber trait except MIC and lint% (in ACGA). It has strong negative correlations (-0.79 in TAGA, -0.80 in ACGA) with UNIF and a weak negative correlation of -0.17 with lint% in TAGA (Table 4).

**TABLE 4:** Correlation between fiber quality traits in crosses between mutant and elite cottons.

	MIC	LEN	UNIF	STR	ELO	SFI%

TAM94L25 (mutants) × GA230						
LEN	−0.30*					
UNIF	0.04	0.39*				
STR	0.04	0.53*	0.44*			
ELO	0	−0.17*	0.28*	–0.07		
SFI%	–0.09	−0.42*	−0.79*	−0.51*	−0.35*	
Lint%	0.19*	−0.11*	0.19*	–0.07	0.59*	−0.17*

**Acala 1517-99 (mutants)** × **GA230**						

LEN	−0.41*					
UNIF	−0.10*	0.49*				
STR	−0.17*	0.58*	0.51*			
ELO	–0.01	−0.15*	0.21*	−0.12*		
SFI%	0.11*	−0.54*	–0.80	−0.55*	−0.26*	
Lint%	0.19*	−0.19*	0.05	−0.23*	0.47*	0.01

*MIC: micronaire, LEN: fiber length, UNIF: uniformity index, STR: fiber strength, ELON: fiber elongation, SFI: short fiber content, lint%: lint percentage.*

*Shows significance at p < 0.0001.

### Genotype and Environmental Effects

ANOVA in both populations showed significant differences between genotypes and environments but no significant interaction between genotypes and environments (Table 5). The variance explained for different fiber traits by genotype ranged from 17 (ELON) to 52% (LEN) in TAGA. Similarly, in ACGA, variance explained by genotype across traits ranges from 21 (ELON) to 56% (LEN).

**TABLE 5:** Analysis of variance components for fiber quality traits in crosses between mutant and elite cottons.

(a) TAM94L25 (mutants) × GA230						

	Source	DF	SS	MS	F Value	%SS explained
MIC	geno	289	111.6	0.39	3.18*	45
	enviro	2	10.73	5.36	44.13*	4
	geno*enviro	576	57.91	0.1	0.83	23
	Error	580	70.48	0.12		
Len	geno	289	2.49	0.01	4.32*	52
	enviro	2	0.09	0.04	21.39*	2
	geno*enviro	576	1.06	0	0.93	22
	Error	580	1.15	0		
UNIF	geno	289	698.67	2.42	1.61*	26
	enviro	2	233.64	116.82	78*	9
	geno*enviro	576	862.2	1.5	1	32
	Error	580	868.64	1.5		
STR	geno	289	2167.71	7.5	2.98*	40
	enviro	2	225.39	112.7	44.76*	4
	geno*enviro	576	1617.44	2.81	1.12	30
	Error	580	1460.24	2.52		
ELON	geno	289	287.58	1	5.24*	17
	enviro	2	1186.71	593.35	3123.91*	70
	geno*enviro	576	106.02	0.18	0.97	6
	Error	580	110.17	0.19		
SFI	geno	289	228.85	0.79	1.59*	27
	enviro	2	86.76	43.38	87*	10
	geno*enviro	576	235.25	0.41	0.82	28
	Error	580	289.22	0.5		
Lint%	geno	289	3998.11	13.83	2.15*	21
	enviro	2	8487.65	4243.82	658.26*	44
	geno*enviro	576	2914	5.06	0.78	15
	Error	580	3739.31	6.45		

**(b) Acala 1517-99 (mutants) × GA230**						

	**Source**	**DF**	**SS**	**MS**	**F Value**	**%SS explained**

MIC	geno	368	106.45	0.29	2.97*	41
	enviro	2	10.3	5.15	52.83*	4
	geno*enviro	734	71.38	0.1	1.00	27
	Error	738	71.94	0.1		
Len	geno	368	3.52	0.01	5.44*	56
	enviro	2	0.05	0.02	13.99*	1
	geno*enviro	734	1.42	0	1.1	23
	Error	738	1.3	0		
UNIF	geno	368	1264.18	3.44	2.55*	34
	enviro	2	415.95	207.97	154.61*	11
	geno*enviro	734	1049.83	1.43	1.06	28
	Error	738	992.75	1.35		
STR	geno	368	4445.48	12.08	4.95*	51
	enviro	2	426.47	213.23	87.46*	5
	geno*enviro	734	2004.12	2.73	1.12	23
	Error	738	1799.23	2.44		
ELON	geno	368	384.75	1.05	5.68*	21
	enviro	2	1177.34	588.67	3196.17*	63
	geno*enviro	734	162.21	0.22	1.2	9
	Error	738	135.93	0.18		
SFI	geno	368	313.33	0.85	2.65*	35
	enviro	2	106.9	53.45	166.12*	12
	geno*enviro	734	231.53	0.32	0.98	26
	Error	738	237.45	0.32		
Lint%	geno	368	6617.96	17.98	3.21*	29
	enviro	2	8614.65	4307.32	768.41*	37
	geno*enviro	736	3624.57	4.92	0.88	16
	Error	738	4136.86	5.61		

*MIC: micronaire, LEN: fiber length, UNIF: uniformity index, STR: fiber strength, ELON: fiber elongation, SFI: short fiber content, lint%: lint percentage.*

*Shows significance at p < 0.0001, geno: genotype, enviro: environment. %SS explained for genotype, environment, and GXE is the ratio of individual sum of squares to total sum of squares.

### Fiber Trait Improvement

#### Improvement for Micronaire and Elongation (Population J)

**FIGURE 2:**
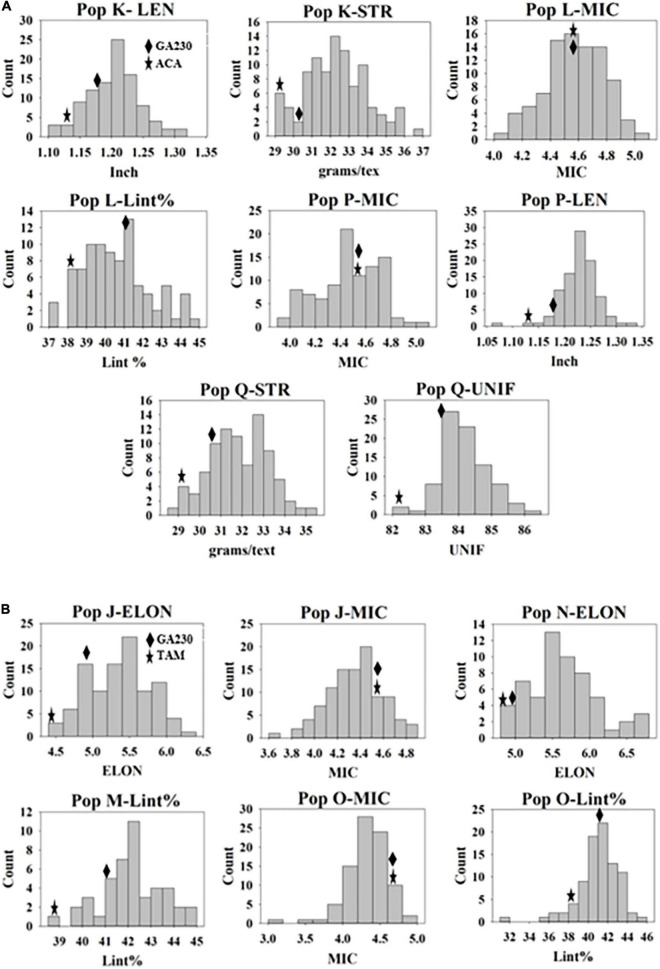
Distribution of genotypes in populations for fiber traits they were developed. **(A)** Populations from Acala 1517-99 (mutant) × GA230. **(B)** Populations from TAM 94L25 (mutant) × GA230. Mean values of M0 parents and GA230 from the data of three environments are indicated on the distribution graph.

**FIGURE 3:**
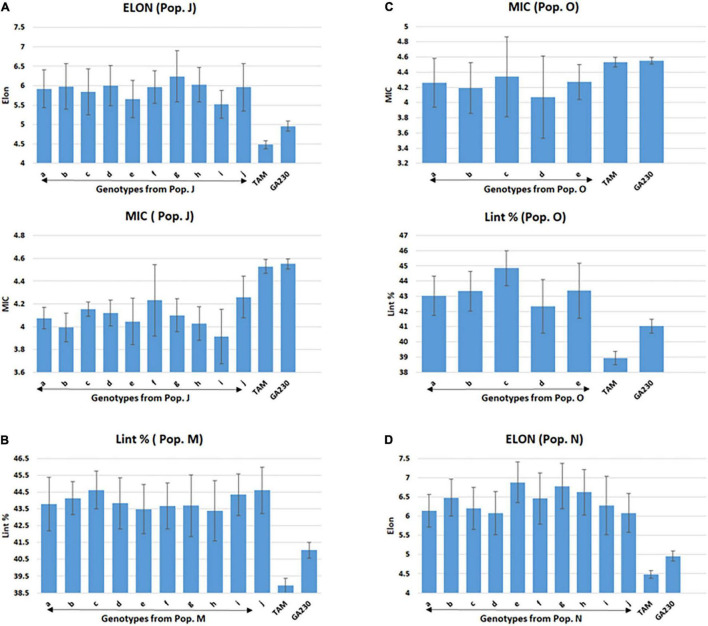
Selected lines showing improvements over parental lines for targeted fiber trait in populations from TAM 94L25 (mutant) × GA230. **(A)** Population J (ELON and MIC), **(B)** population M (Lint %), **(C)** population O (MIC and Lint%), and **(D)** population N (ELON). Common letters on the x-axis of the graph indicate the same genotype of pops J (graphs A) and O (graphs C). Error bar indicates a standard error (SE).

A total of 100 lines were evaluated from “population J,” placing TAM 94L25 mutants for improved MIC and ELON in GA230 background, with overall means that showed significant (*p* < 0.01) improvement over the parental lines and exceeded the midparent values by 4% (MIC) and 13.6% (ELON) (Table 6). A significant improvement over the parents was realized for MIC by 30 lines with a maximum improvement of 19% over the midparent value; and for ELON by nine lines with a maximum improvement of 32.2% over the midparent value (Figure 2 and Table 6). The top 10 lines based on the sum of Z scores reflecting enhanced UNIF and STR showed an average improvement of 9.9% in MIC and 25.2% in ELON relative to midparent values, and each significantly improved (Figure 3).

**TABLE 6:** Comparison between parentals or checks and populations from crosses between elite cotton line GA230 and mutants for fiber quality traits.

Population id	Trait	Population mean	Bkgrd (Acala or TAM)	GA230	Midparent (Bkgrd + GA230 avg.)	% Improvement over midparent	Fiber max 832	Delta pine 393	Total # of improved lines in population	% Improvement of best line to mid-parent

Acala 1517-99 (mutants) × GA230										
K	LEN	1.21	1.12*	1.17*	1.15	5.7	1.14*	1.13*	40	15.8
K	STR	32.31	29.15*	30.48*	29.82	8.4	30.6*	30.3*	54	22.4
L	MIC	4.57	4.54	4.55	4.55	0.6	4.26	4.57	7	-10.4
L	Lint%	40.56	38.01*	41.04	39.53	2.6	40.2	39.5	2	13.1
P	LEN	1.23	1.12*	1.17*	1.15	7.4	1.14*	1.13*	67	16.1
P	MIC	4.46	4.54	4.55	4.55	-1.9	4.26	4.57	18	-12.8
Q	STR	31.83	29.15*	30.48*	29.82	6.8	30.6*	30.3*	36	17.9
Q	UNIF	84.18	82.11*	83.6*	82.86	1.6	83*	82.7*	15	4.1

**TAM94L25 (mutants)** × **GA230**										

J	ELON	5.36	4.48*	4.96*	4.72	13.6	5.11	5.28	9	32.2
J	MIC	4.36	4.53*	4.55*	4.54	-4	4.26	4.57*	30	-19
M	Lint%	42.2	38.93*	41.04	39.99	5.5	40.2*	39.5*	2	11.6
N	ELON	5.66	4.48*	4.96*	4.72	19.9	5.11*	5.28	11	45.8
O	MIC	4.32	4.53*	4.55*	4.54	-4.8	4.26	4.57*	25	-31.7
O	Lint%	41.13	38.93*	41.04	39.99	2.9	40.2	39.5*	3	13.9

Parental or check cells with “*” are significantly inferior to population average by p < 0.01. MIC: micronaire, LEN: fiber length, UNIF: uniformity index, STR: fiber strength, ELON: fiber elongation, lint%: lint percentage.

#### Improvement for Length and Strength (Population K)

**FIGURE 4:**
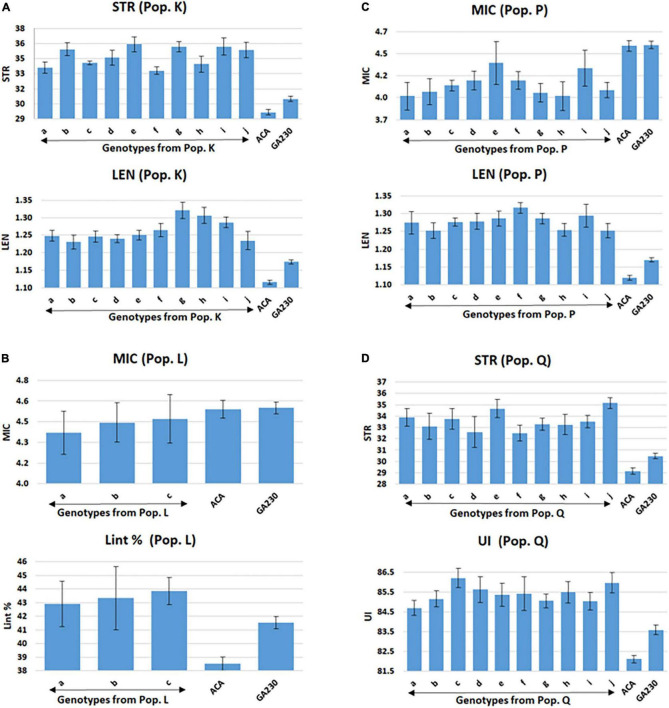
Selected lines showing improvements over parental lines for both targeted fiber traits in populations from ACALA1517-99 (mutant) × GA230. **(A)** Population K (LEN and STR), **(B)** population L (MIC and Lint %), **(C)** population P (MIC and LEN), and **(D)** population Q (STR and UNIF). Common letters in x-axis of the graph indicate same genotype of populations K (graphs A), L (graphs B), P (graphs C), and Q (graphs D). Error bar indicates a standard error (S.E).

A total of 98 lines were evaluated from “population K,” placing Acala 1517-99-derived mutants for LEN and STR in GA230 background, with overall means that showed significant improvement (*p* < 0.01) over the parental lines and exceeded the midparent values for LEN by 5.7% and STR by 8.4% (Table 6). A significant improvement over the parents was realized for LEN by 40 lines with a maximum improvement of 15.8%; and for STR by 54 lines with a maximum improvement of 22.4% (Figure 2 and Table 6). A total of 10 lines based on the sum of Z scores reflecting enhanced LEN and STR significantly exceeded both parents and showed improvements over the midparent values of 10.7% in LEN and 16.6% in STR (Figure 4).

#### Improvement for Micronaire and Lint% (Populations L and O)

A total of 89 and 87 lines were evaluated from “population L” and “population O,” placing Acala 1517-99 and TAM 94L25-derived mutants, respectively, for MIC and lint% into GA230 background. The average of individuals in population O showed significant improvement for MIC compared to parental lines, while there was no significant difference between population L and parents. For lint%, means of both populations L and O were significantly better than their respective mutant sources (Acala 1517-99 or TAM 94L25), but not exceeding GA230. A significant improvement over parents was realized for MIC by 25 and seven lines in populations O and L, respectively. With respect to midparent MIC value, the most positive changes were 31.7% (population O) and 10.4% (population L). For lint%, a significant improvement over the parents was noticed in three lines in population O with a maximum gain of 13.9%, and in two lines in population L with a maximum advancement of 13.1%. Due to the negative association between MIC and lint%, it was difficult to obtain lines with simultaneous improvement for both traits. Only five lines showed promise for improving both MIC and lint% in population O. The MIC mean of these lines was 8.2% lower (better) than the midparent value and the lint% mean was 6.8% higher than midparent value (Figure 3).

#### Improvement for Lint% (Population M) and Elongation (Population N)

Two populations were developed to improve lint% (population M), Elon (population N), and Rd value (both) in GA230 background. Mutant lines M276 (lint%), M1251 (Rd), and M2925 (Elon) used in the breeding scheme were from TAM 94L25 background. Unfortunately, measurements of Rd value were not obtained, so no pyramiding was further considered for these populations. Based on the performance in 2013 for lint% (in population M) and Elon (in population N), only selected progenies from population M (45 lines) and population N (58 lines) were further evaluated.

The average lint% of population M was significantly higher than TAM 94L25 but not GA230. Only two lines were significantly improved over both parents. The top 10 lines for lint% in population M showed an average 9.9% improvement over the midparent value (Figure 3).

The average ELON of population N was 19.9% higher than the midparent value and significantly better than both parents. A total of 11 lines showed improvement over parents, with a maximum improvement of 45.8% higher than the midparental value. The top 10 lines in the population for ELON showed an average 35.6% improvement over the midparental value (Figure 3).

#### Improvement for Micronaire and Length (Population P)

Population P was developed by crossing Acala 1517-99-derived mutant for LEN and MIC with GA230, and a total of 97 lines of the population were evaluated for fiber traits. The average mean of LEN in the population showed significant improvement (*p* < 0.01) over the parental lines and exceeded the midparent values for LEN by 7.4%, while MIC showed no significant improvement over parental lines. A significant improvement over the parents was realized for LEN by 67 lines with a maximum improvement of 16.1%; and for MIC by 18 lines with a maximum improvement of 12.8% (Figure 2 and Table 6). In total, based on the sum of Z scores reflecting enhanced LEN and MIC, ten lines significantly exceeded both parents and showed improvements over the midparent values of 12% in LEN and 9.6% in MIC (Figure 4).

#### Improvement for Strength and Uniformity Index (Population Q)

A total of 87 lines were evaluated from “population Q,” placing Acala 1517-99-derived mutants for STR and UNIF in GA230 background, with overall means that showed significant improvement (*p* < 0.01) over the parental lines and exceeded the midparent values for STR by 6.8% and UNIF by 1.6% (Table 6). A significant improvement over the parents was realized for STR by 36 lines with a maximum improvement of 17.9%; and for UNIF by 15 lines with a maximum improvement of 4.1% (Figure 2 and Table 6). Based on the sum of Z scores reflecting enhanced LEN and STR, a total of 10 lines significantly exceeded both parents and showed improvements over the midparent values of 3.1% in UNIF and 12.6% in STR (Figure 4).

## Discussion

Building on a few prior efforts that have produced improved cotton germplasm lines through EMS-generated mutations (Auld et al., 2000; Bechere et al., 2009; Brown et al., 2012; Patel et al., 2014, 2016) from a total of 157 lines validated as affecting quantitative fiber quality parameters (Patel et al., 2014), here we further evaluated mutant alleles of nine striking mutants, confirming improvement for fiber properties in a new elite genetic background (Table 6). Further, comparison to two commercial checks (Deltapine 393 and Fibermax 832) showed mutants to be superior in several cases, including means of STR and LEN of population K, STR and UNIF of population Q, lint% of population M, and LEN of population P (Table 6). These findings provide additional strong support to the value of these putatively novel alleles for improving fiber qualities of elite cotton.

Each of the eight test populations in the study was based on intercrossing of two F1s that involved different fiber quality mutants. All populations contained multiple genotypes improved for at least one of two target traits; and from the four populations (J, K, P, and Q), the average of 10 lines selected based on summed Z scores for the two traits was better for both traits than the parental lines (Figure 4), providing a natural foundation for pyramiding of fiber quality traits.

Based on the results of Populations O and L, it seems difficult to simultaneously improve two traits like MIC and lint% that have a negative association. Still, this conclusion is based on only four mutant lines, two for MIC (one from Acala 1517-99 and one from TAM 94L25) and two for lint% (1,1) from a large set of improved mutant lines for lint% (7, 10) and MIC (18, 5) (Patel et al., 2014). Testing more combinations of improved mutant lines might identify alleles that reduce the negative association of lint% and MIC. Development of higher STR and higher yielding lines was possible despite the traditional negative association between STR and yield, suggesting that this association might be due to the linkage between genes rather than pleiotropic effects that cause this negative correlation (Culp et al., 1979; Zhang et al., 2003). On the other hand, crosses for traits having a positive correlation, like LEN and STR (population K), LEN and MIC (population P), and STR and UNIF (population Q), produced lines that were significantly improved for both traits. Furthermore, traits like MIC and ELON that had no correlation in our population were improved simultaneously in population J, which was developed targeting these traits (Figure 4).

Within a population, the number of lines showing significant improvement for a trait relative to both parents ranges from two (lint% in population L) to 64 (LEN in population P) (Table 6). Populations L and P were developed from a common mutant line Acala 1517-99-M3010 for MIC and Acala 1517-99-M1524 (population L for lint%) or Acala 1517-99-M1903 (population P for LEN). One reason for this variation may be that yield components are controlled by a more complex genetic network than fiber quality components. It might also be possible that GA230, elite germplasm, has less scope to improve lint% than LEN. Another factor might be that some populations combine negatively associated traits like lint% and MIC, such as population L. The effect of the cross can also be seen on the MIC of population L. Mutant line Acala 1517-99-3010 for MIC was used to develop both populations P and L, showing 18 (18.6%) improved lines in population P but only 7 (8%) in population L.

Populations J and N were developed with the intention to improve ELON based on mutant line TAM 94L25-2925 which had previously shown 50% improvement over the control lines. Compared to midparental values, the top 10 lines showed an average gain of 27.6% and 35.6% for ELON in population J and N, respectively, with the best lines showing 32.2% and 45.8% improvement, respectively. The result suggests considerable scope for improving ELON in elite germplasm such as GA230. This might be because ELON has only recently become a priority in cotton breeding and therefore has a short history of selection in elite germplasm (Zhang et al., 2011).

Many lines showed remarkable improvement for additional fiber traits beyond those known to be conferred by the mutants used in population development. Such effects might be due to a strong positive correlation between traits; thus mutants identified for single fiber traits might also contribute to the improvement of other traits. Similar results were seen in the pilot study for mutant lines 3010 and 1903 from Acala 1517-99 background (Patel et al., 2014). Lines that combine multiple desirable traits may warrant release as germplasm after verifying the stability of the phenotype and determining their yield potential, as done by previous studies (Bechere et al., 2007, 2011).

In summary, we validated that novel EMS-derived alleles confer improved fiber quality to elite cultivars beyond those in which they were identified and can be combined to obtain improved genotypes for multiple fiber traits. Populations segregation for multiple mutations can be used to develop new germplasm and to map novel alleles conferring improved fiber quality, accelerating progress by using high-quality cotton genome sequences (Paterson et al., 2012; Li et al., 2015) and contemporary molecular techniques like genotype-by-sequencing (GBS) which can provide plentiful numbers of SNPs (Davey et al., 2011). The availability of the cotton genome sequence will further support the process (Paterson et al., 2012; Li et al., 2015). Moreover, the 157 different mutant lines (103 Acala 1517-99 and 54 TAM 94L-25) that were superior to parental or control lines in fiber traits including LEN, uniformity, strength, fineness, elongation, Rd value, and in some cases multiple attributes (Patel et al., 2014) provide rich scope for further improvements beyond the alleles studied here, and may not represent saturation of the potential alleles that can be EMS-mutagenized or engineered into elite germplasm using genome editing techniques such as CRISPR/CAS9 to improve fiber traits without disturbing any other region in the genome.

## Data Availability Statement

The raw data supporting the conclusions of this article will be made available by the authors, without undue reservation.

## Author Contributions

JP developed the population, performed the experiment, conducted the analysis, and drafted and revised the manuscript. SK, RC, JA, NB, and PC helped in planting, agronomical practices, and collecting phenotypic data. DJ analyzed fiber samples. AP conceived the project, acquired the funds, supervised, conducted the analysis, and drafted and revised the manuscript. All authors contributed to the article and approved the submitted version.

## Conflict of Interest

The authors declare that the research was conducted in the absence of any commercial or financial relationships that could be construed as a potential conflict of interest.

## Publisher’s Note

All claims expressed in this article are solely those of the authors and do not necessarily represent those of their affiliated organizations, or those of the publisher, the editors and the reviewers. Any product that may be evaluated in this article, or claim that may be made by its manufacturer, is not guaranteed or endorsed by the publisher.

## Footnotes

1
https://www.cottoninc.com/
